# Microarray Profiling and Co-Expression Network Analysis of LncRNAs and mRNAs in Neonatal Rats Following Hypoxic-ischemic Brain Damage

**DOI:** 10.1038/srep13850

**Published:** 2015-09-09

**Authors:** Fengyan Zhao, Yi Qu, Junyan Liu, Haiting Liu, Li Zhang, Yi Feng, Huiqing Wang, Jing Gan, Ruifeng Lu, Dezhi Mu

**Affiliations:** 1 Department of Pediatrics, West China Second University Hospital, Sichuan University, Chengdu 610041, China; 2 Key Laboratory of Obstetric & Gynecologic and Pediatric Diseases and Birth Defects of Ministry of Education, Sichuan University, Chengdu 610041, China; 3 Department of Pediatrics, Binzhou Medical University Hospital, Binzhou 256600, China; 4 Department of Pediatrics, University of California, San Francisco, CA94143, USA

## Abstract

Long noncoding RNAs (lncRNAs) play critical roles in cellular homeostasis. However, little is known about their effect in developing rat brains with hypoxic-ischemic brain damage (HIBD). To explore the expression and function of lncRNA in HIBD, we analyzed the expression profiles of lncRNAs in hypoxic-ischemic (HI) brains and sham control using microarray analysis. The results showed a remarkable difference in lncRNA between HI and sham brains. A total of 322 lncRNAs were found to be differentially expressed in HI brains, compared to sham control. Among these, BC088414 was one of the most significantly urpregulated lncRNAs. In addition, 375 coding genes were differentially expressed between HI brains and sham control. Pathway and gene ontology analysis indicated that the upregulated coding genes mostly involved in wounding, inflammation and defense, whereas the downregulated transcripts were largely associated with neurogenesis and repair. Moreover, coding non-coding co-expression network analysis showed that the BC088414 lncRNA expression was correlated with apoptosis-related genes, including Casp6 and Adrb2. Silencing of lncRNA BC088414 in PC12 cells caused reduced mRNA level of Casp6 and Adrb2, decreased cell apoptosis and increased cell proliferation. These results suggested lncRNA might participate in the pathogenesis of HIBD via regulating coding genes.

Hypoxic ischemic encephalopathy (HIE) remains an important cause of neonatal morbidity and mortality^1^. With advances in obstetrical and neonatal care, the survival rate of HIE has improved. Consequently, the numbers of infants who are at risk for the permanent neurological deficits has also increased^1^. Therefore, HIE is still a considerable threat to children’s health and lasting quality of life and further work are needed for effective neurotherapeutic interventions for HIE.

Long noncoding RNAs (lncRNAs) are currently thought to have vital and wide-raging functions, including mediating local and higher-order epigenetic states and modulating posttranscriptional RNA processing, transport, stabilization, metabolism, and translation^2^,^3^. LncRNAs are particularly abundant in the brain and play vital roles in mediating many aspects of brain evolution, development, homeostasis, and plasticity^4^,^5^. Moreover, increasing evidence suggests that altered expression and misregulation of lncRNAs can both result in and modulate the pathophysiology of a spectrum of neurological and psychiatric disorders^6^,^7^,^8^,^9^,^10^. Therefore, analysis of lncRNAs may serve to broaden our understanding of the molecular mechanisms of HIE, providing impetus for identifying new therapeutic targets.

Previous studies in the adult rat have shown that after stroke, cerebral lncRNA expression profiles are extensively altered and contribute to the stabilization of mRNA expression^11^. Few studies to date, however, have evaluated lncRNA changes in developing brain after HI injury. To the best of our current knowledge, methods for the identification of novel disease-related lncRNAs includes microarray, qRT-PCR, RNA-seq and capture sequencing. In this study, we adopted a microarray analysis to detect the lncRNAs expression in developing brain following HIBD. Our results revealed that the expression profile of lncRNAs is dramatically different between HI brains and sham control. The lncRNA BC088414 is upregulated in HI brains. Additionally, we constructed a co-expression network of lncRNAs and coding gene transcript and found a correlation of BC088414 to 3 apoptosis-related genes, indicting a potential co-regulation function of lncRNA BC088414. In particular, silencing of lncRNA BC088414 attenuated HI injury with decreased levels of Casp6 and Adrb2.

## Results

### Overview of lncRNA profiles in neonatal rat model of HIBD

**Figure 1:**
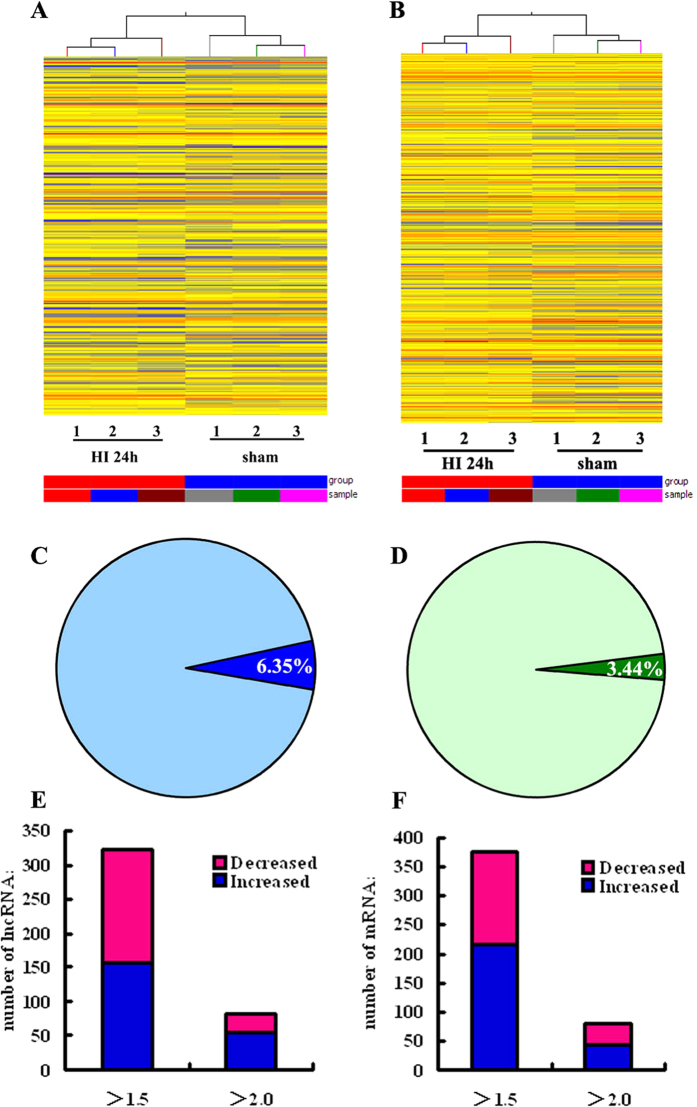
Differentially expressed lncRNAs and mRNAs between neonatal brains at 24 h after HI and sham treatment. (**A,B**) Hierarchical clustering of lncRNAs (**A**) and mRNAs (**B**) differentially expressed in neonatal brains at 24 h following HI (HI 24 h) or sham treatment. The intensity of the color scheme corresponds to the log2 expression values. Red indicates high relative expression, and blue indicates low relative expression. (**C,D**) Distribution of differentially expressed transcripts (dark colors) in HI 24 h brains for lncRNAs (**C**) and mRNAs (**D**). Lighter colors refer to the total number of transcripts, and darker colors refer to the number of differentially expressed transcripts. (**E,F**) Column chart of differentially expressed transcripts in HI 24 h brains for lncRNAs (**E**) and mRNAs (**F**). Bar plots show the number of up-regulated transcripts (blue) or down-regulated transcripts (pink) at different fold change cut-off values.

To profile differentially expressed lncRNAs in developing brains with HIBD, we generated a neonatal rat model of HIBD using the modified Rice-Vannucci method. At 24 h after HI injury, the animals were executed for microarray analysis of lncRNA expression. Variations in lncRNA expression levels are shown in Fig. 1 and Table 1. A total of 5,067 lncRNAs was detected in the neonatal rat brain, 322 of which (6.35%) responded to HI brain injury with a fold change of at least 1.5 (*P *< 0.05, Fig. 1A,C and Table 1 ). Among the 322 differentially expressed lncRNAs in HI brains, 157 lncRNAs were upregulated and 165 lncRNAs were downregulated (*P* < 0.05, Fig. 1E and Table 1). 82 lncRNAs exhibited a high fold change of at least 2-fold, where 54 lncRNAs exhibited increased expression and 28 lncRNAs decreased expression (Table 1). MRAK010747 (fold change: 4.741, *P* < 0.05) was the most upregulated lncRNA. In addition, BC088414 was one of upregulated lncRNAs in HI brains compared with sham control (fold change: 1.675, *P* < 0.01).

### Overview of coding gene profiles in neonatal rat model of HIBD

The microarray analysis also comprised the expression profile of coding genes. A total of 10904 coding transcripts (mRNA) were detected in developing brains, 375 out of which, approximately 3.44%, were differentially expressed (>1.5-fold, *P* < 0.05) (Fig. 1B,D and Table 1). Among the 375 deregulated mRNAs, 215 mRNAs were upregulated and 160 mRNAs were downregulated (*P* < 0.05, Fig. 1F and Table 1). A total of 81 mRNAs exhibited a high fold change of at least 2-fold, and the proportion of increased and decreased mRNAs was approximately 50/50 (Fig. 1F and Table 1). Hierarchical clustering analysis was used to group mRNAs based on their expression levels (Fig. 1B).

### Validation of deregulated lncRNAs in neonatal brains after HI treatment

**Figure 2:**
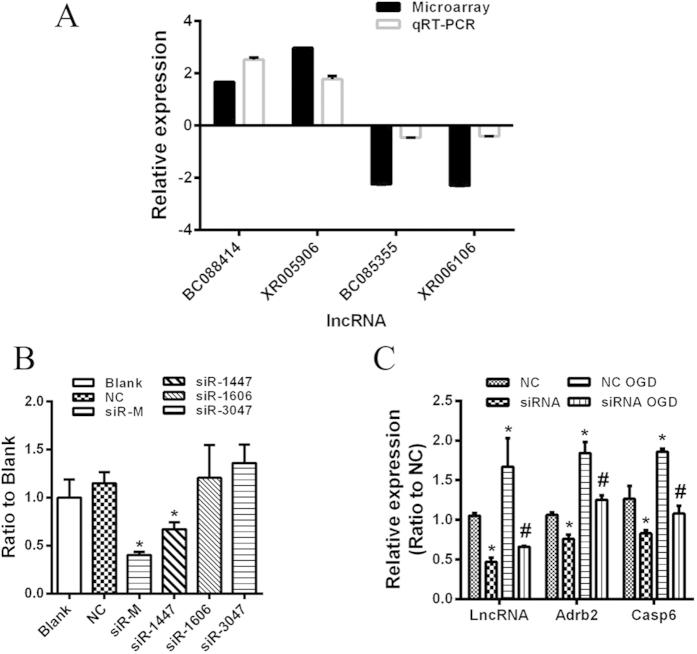
Gene expressions. (**A**) Comparison between microarray and qRT-PCR results. Four differentially expressed lncRNAs were validated by qRT-PCR. The columns in the chart represent the absolute fold-change between the groups (HI/sham) detected by microarray and qRT-PCR. The bars represent standard errors. The qRT-PCR results were closely correlated with the microarray data. *P < 0.05. (**B**) Knockdown efficiency of BC088414 specific siRNAs in PC12 cells. siRNA-1447 (siR-1447) and combination of all 3 siRNAs (siR-M) significantly inhibited BC088414 expression. *P < 0.05 versus Blank group. (**C**) Inhibition of BC088414 reduced expression of Adrb2 and Casp6 in PC12 cells following OGD treatment. The expression of BC088414, Adrb2 and Casp6 were overexpressed in PC12 cells after OGD. Knockdown of BC088414 abated the increase of Adrb2 and Casp6 in OGD treated PC12 cells. *P < 0.05 versus NC group. #P < 0.05 versus NC OGD group. Blank: non-transfected group; NC: nonsense siRNA transfected group; OGD: oxygen and glucose deprivation; NC OGD: nonsense siRNA transfected PC12 cells following OGD; siRNA OGD: siR-M transfected PC12 cells following OGD.

Four lncRNAs were selected for validation of the microarray results using qRT-PCR. Consistent with the microarray data, qRT-PCR analysis revealed that the expression of BC088414 and XR005906 was upregulated, whereas BC085355 and XR006102 were downregulated in neonatal HI brains (Fig. 2A and Table 2). Therefore, the qRT-PCR results confirmed the accuracy of microarray findings. These findings provide confirmatory evidence that lncRNA BC088414 is upregulated in developing brains after HI injury.

### GO analysis

**Figure 3:**
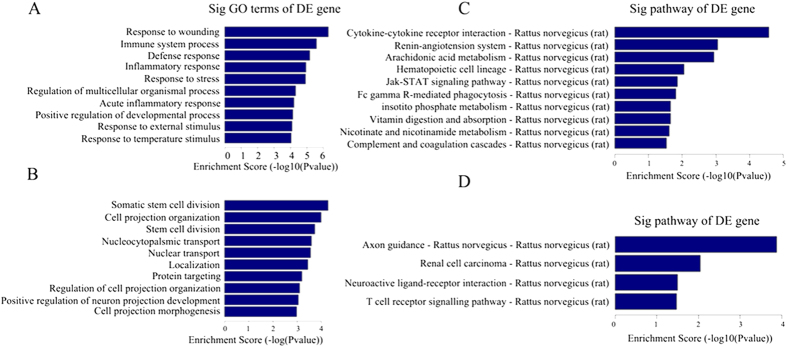
GO enrichment and KEGG pathway analysis of differentially expressed genes in HI brains according to biological processes. (**A**) Top 10 GO terms enriched among upregulated genes. (**B**) Top 10 GO terms enriched among downregulated genes. (**C**) The top 10 pathways enriched among the upregulated mRNAs in HI brains. (**D**) All pathways enriched among the downregulated mRNAs in HI brains. The bar plot depicts the enrichment scores. The *P* value indicates the significance of the correlation between the pathway and HIBD.

It is widely accepted that lncRNAs can regulate the expression of adjacent or overlapping protein-coding genes. To this end, the function of lncRNAs may be reflected in their associated protein-coding genes^3^. Therefore, GO enrichment analysis of differentially expressed mRNAs may provide insight into the function of these differentially expressed lncRNAs. GO analysis covers the following 3 domains: cellular components, biological processes and molecular functions. With respect to biological processes, the highest enriched GO terms targeted by upregulated transcripts included the response to wounding, immune system process, defense response, inflammatory response and response to stress (Fig. 3A). The highest enriched GO terms targeted by downregulated transcripts included somatic stem cell division, cell projection organization, nucleocytoplasmic transport, nuclear transport and localization (Fig. 3B). Importantly, the GO terms somatic stem cell division and cell projection organization were associated with ASPM, which is critical for proper neurogenesis and neuronal migration^12^. Interestingly, our results suggested that the upregulated transcripts related to biological processes were specific to wounding, inflammation and defense, whereas the downregulated transcripts were largely associated with neurogenesis and repair, aptly corresponding to the pathophysiology of HIBD in neonatal rats.

### Pathway analysis

Pathway analysis of differentially expressed mRNAs is designed to provide insight into the cell pathways associated with these genes. Our results demonstrated that 18 pathways corresponded to upregulated transcripts and 4 to downregulated transcripts (Fig. 3C,D). Cytokine-cytokine receptor interaction was the top pathway enriched among upregulated protein-coding genes, and axon guidance was the top pathway enriched among downregulated genes. This suggests that these pathways may harbor significance and/or may contribute to the pathogenesis and biochemical characteristics of HIBD in neonatal rats.

### Expression of BC088414 correlates with coding genes

**Figure 4:**
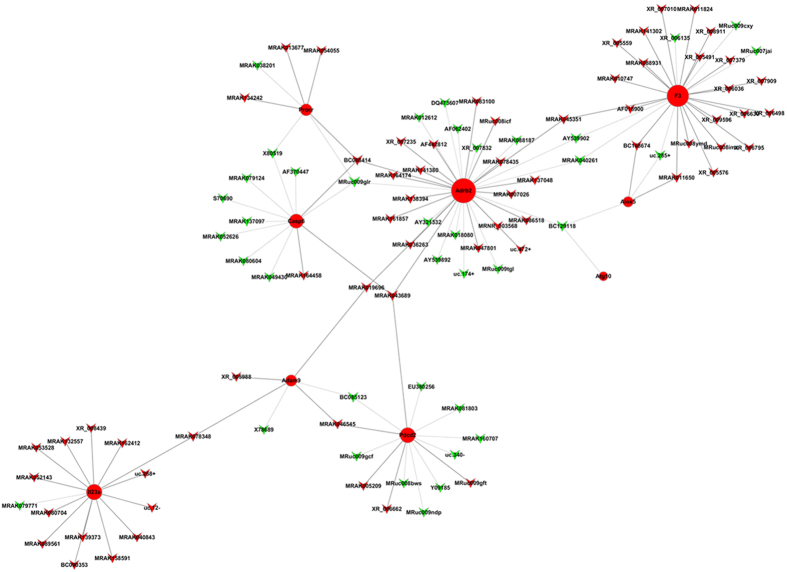
Coding non-coding gene co-expression networks of the nine differentially expressed mRNAs. The network represents co-expression correlations between the nine mRNAs and significantly differentially expressed lncRNAs. Nodes represent coding genes, and circles represent lncRNAs. Red indicates upregulated genes and green indicates downregulated gens. Solid lines indicate positive correlations, and dashed lines indicate negative correlations.

We selected 9 significantly upregulated coding genes in HI brains to construct a coding noncoding gene co-expression network (CNC). These coding genes are involved in multiple biological processes, including cell death, apoptosis and defense response. The CNC network showed that lncRNA BC088414 was positively correlated with Procr, Casp6 and Adrb2 (Fig. 4), which are all correlated with apoptosis. According to these results, we focused on lncRNA BC088414 for further functional studies to elucidate its role in pathogenesis of HIBD.

### Knockdown of BC088414 reduces expression of Casp6 and Adrb2 in OGD treated PC12 cells

Considering the feasibility and transfection efficiency, we adopted an oxygen-glucose deprivation (OGD) model with PC12 cells for function research of lncRNA in cerebral HI injury. Three different siRNAs were transfected individually or in combination (siR-M) into PC12 cells to inhibit BC088414 expression. The efficacy of siRNA transfection was detected by qRT-PCR. It confirmed that BC088414 expression was significantly reduced after transfection of siR-M and siRNA-1447 (siR-1447) (Fig. 2B). In particular, siR-M could reduce by >50% the BC088414 level, compared with the non-transfected group (Blank) (*P* < 0.05, Fig. 2B). Nonsense siRNA (NC), siRNA-1606 (siR-1606), and siRNA-3047 (siR-3047) had no effect on BC088414 expression (Fig. 2B). Therefore, we employed siR-M to inhibit BC088414 expression for subsequent functional analysis of BC088414.

After OGD, BC088414 expression was dramatically upregulated, and the level of Casp6 and Adrb2 mRNA was sharply increased. SiRNA treatment significantly suppressed the increase of BC088414 expression induced by OGD (*P* < 0.05, Fig. 2C). As a consequence of the inhibition, the elevation of mRNA for Casp6 and Adrb2 was inhibited (*P* < 0.05, Fig. 2C). These findings suggested that the lncRNA BC088414 could regulated the level of mRNA Casp6 and Adrb2.

### Knockdown of BC088414 inhibits cell proliferation and promotes apoptosis of PC12 cells exposed to OGD

**Figure 5:**
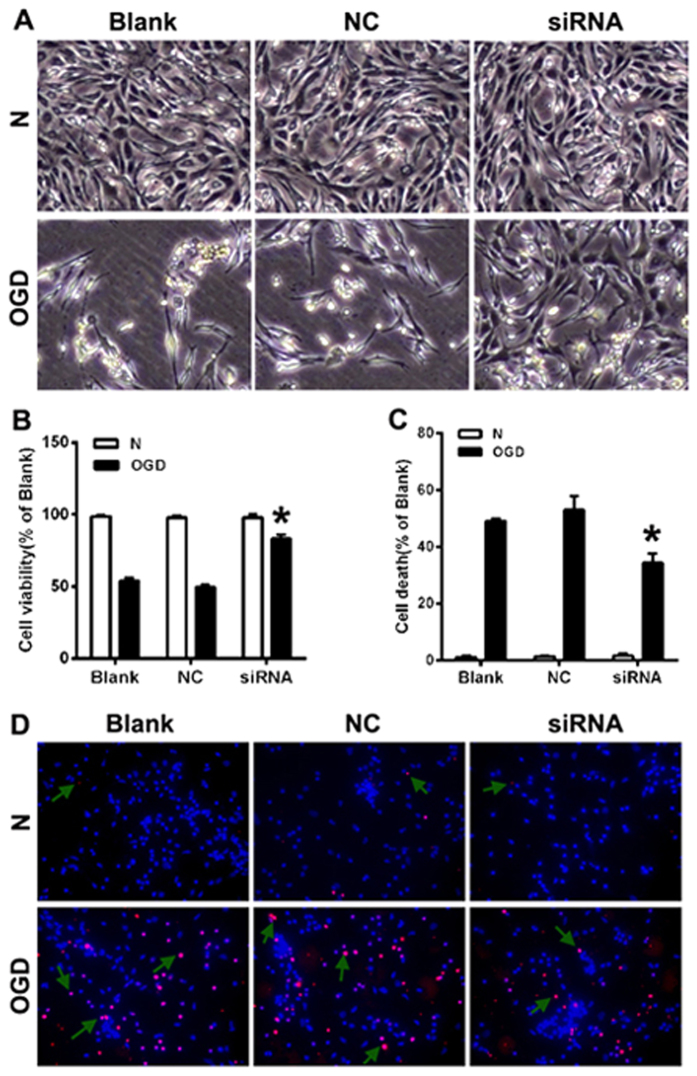
Silencing of BC088414 promoted proliferation and suppressed apoptosis in OGD treated PC12 cells. (**A**) Morphological observation of cell growth state. Following OGD, the numbers of PC 12 cells were reduced. Compared with Blank and NC groups, the number of living cells in siRNA group was increased. (**B**) CCK8 cell proliferation assay in PC 12 cells. The cell viability of PC12 cells was decreased after OGD. SiRNA transfection induced higher growth rate compared to Blank and NC. (**C,D**) TUNEL staining in PC12 cells. The apoptosis cells were increased after OGD. Knockdown of BC088414 in PC12 cells reduced apoptosis induced by OGD. Arrows shows positive staining cells. *P < 0.05 versus Blank group. Blank: non-transfected group; NC: nonsense siRNA transfected group; siRNA: siRNA transfected PC12 cells; N: normoxia; OGD: oxygen and glucose deprivation.

We examined whether knockdown of BC088414 influences cells’ resistance against OGD by morphological observation and CCK8 assay. Compared with Blank and nonsense siRNA (NC) transfected group, cell death induced by OGD in BC088414 siRNA treated group was dramatically lessened (*P* < 0.05, Fig. 5A) as evidenced by morphological observation. CCK8 assay showed that silencing of BC088414 promoted cell proliferation after OGD, whereas NC did not evidently affect cell viability during OGD (Fig. 5B). These results seemed to suggest that knockdown of BC088414 protected cells against OGD injury. Meanwhile, silencing of BC088414 attenuated cell apoptosis induced by OGD as demonstrated by TUNEL staining (*P* < 0.05 *v.s.* the Blank group, Fig. 5C,D). All these findings implicated that knockdown of BC088414 attenuated apoptosis and strengthened cells’ resistance against OGD.

## Discussion

In the present study, we evaluated the cerebral expression of lncRNAs in both HI-injured neonatal rats and sham controls using microarray analysis. There were 5,067 lncRNAs detected, 322 of which were significantly differentially expressed. In particular, BC088414 was upregulated in neonatal cerebral after HI. Furthermore, knockdown of BC088414 inhibited overexpression of Adrb2 and Casp6, decreased cell apoptosis and increased cell proliferation. Collectively, these findings showed that cerebral HI injury influenced lncRNA expression in neonatal rats and that altered lncRNA expression might exert effects on the development and progress of HIBD via regulating mRNA expression.

The inflammatory response, in conjunction with excitotoxic and oxidative responses, is the major contributor to ischemic injury in both the immature^13^ and adult brain^14^. Within minutes of an insult to the neonatal brain, both astrocytes and microglia are activated^15^,^16^, cytokines and chemokines are produced in excess, and activated leukocytes migrate to the site of injury, all of which collectively result in delayed neuronal death, axonal degeneration and breakdown of the immature blood-brain barrier (BBB)^15^,^17^. In line with these results, GO analysis revealed that the upregulated genes at 24 h after HI were mainly enriched for GO terms associated with the response to wounding, immune system processes, defense responses and inflammatory responses. Pathway analysis also indicated that inflammation-associated pathways, such as cytokine-cytokine receptor interaction pathways, were the most enriched pathways targeted by the upregulated genes. This particular category included 10 genes, including CXCL10 and TNF, etc. Tumor necrosis factor-α (TNF-α) is a pro-inflammatory cytokine exerting both homeostatic and pathophysiological roles in the central nervous system^18^. Consistent with our findings, it has been demonstrated that CXCL10 and TNF-α mRNAs are profoundly upregulated in the 24 hours after transient middle cerebral artery occlusion (tMCAO)^19^,^20^.

Neurogenesis is important for neurological recovery following cerebral ischemia^21^,^22^, and mounting evidence shows that HI injury stimulates neurogenesis^23^,^24^. However, this phenomenon has only been reported after a latent period of at least 7 days of recovery^23^,^25^, and few data are available regarding neurogenesis at the early stage of recovery after HI. Our results indicated that genes enriched for GO terms related to somatic stem cell division, cell projection organization and stem cell division were downregulated at 24 h after HI, indicating that neurogenesis may be suppressed in ischemic brains. Furthermore, this delay in neurogenesis may be attributed to excessive inflammation at 24 h post-HI, as it has been demonstrated that exposure to recombinant IL-6 or TNF-α decreases neurogenesis *in vitro* by approximately 50%^26^. Thus, it is possible that the IL-6 and TNF-α released by activated microglia following cerebral HI injury prevents neurogenesis. In addition, the microvasculature of the hippocampus is a critical element of the neurogenic microenvironment^27^, and HI damages the microvasculature^28^ at both early and progressive time points in the neonatal brain.

Pathway analysis also revealed that the axon guidance pathway was the most enriched pathway targeted by downregulated genes. This particular group included 6 genes, DCC, MET, NRP1, PAK3, PAK6, and UNC5B, all of which exhibited decreased expression. DCC (deleted in colorectal cancer) and UNC5B are two main receptors for netrins^29^. Netrins are a family of proteins that mediate axon guidance and direct axon migration during embryogenesis^29^ and can promote the recovery of neuronal function after cerebral ischemia^30^,^31^,^32^. These results are highly consistent with those of our GO analysis; supporting the notion that neurogenesis is blocked at an early stage of recovery after HI.

Based on data from our GO analysis, we constructed a CNC network to further analyze the correlation of lncRNAs and deregulated mRNAs. It has been suggested that lncRNAs affect transcription of proximal or distal genes via cis- and trans-acting mechanisms^33^. We also observed that the lncRNA BC088414 was positively correlated with Procr, Adrb2 and Casp6 (Fig. 5), especially correlated to Adrb2 and Casp6 (PCC > 0.90). Both Adrb2^34^ and Casp6^35^ are involved in cell death and found to be important following stroke or hypoxia-ischemia. Collectively, we speculated that BC088414 may regulate the expression of these two genes and correlate with cell death induced by cerebral hypoxia-ischemia. To confirm this hypothesis, the expression of BC088414 in PC12 cells was knocked down using small interfering RNA (siRNA). 24 hours after siRNA transfection, PC12 cells were exposed to OGD treatment. Silencing of BC088414 resulted in reduced expression of Adrb2 and Casp6. Moreover, inhibition of BC088414 aslo increased cell proliferation and decreased cell apoptosis. Clearly, these findings suggested that BC088414 is a functional lncRNA in and may contributed to cell death in OGD treated PC12 cells via interaction with Adrb2 and Casp6. Taking together, our findings implicated that BC088414 may involve in neuronal survival in cerebral following HI injury. More exploration needed to identify the detailed mechanisms and biological functions of lncRNAs in HIBD.

## Conclusion

In conclusion, our study demonstrated that HI injury altered the expression profiles of lncRNAs in the neonatal rat brain. Particularly, BC088414 was upregulated after HI. Knockdown of lncRNA BC088414 attenuated cell apoptosis and promoted cell proliferation in PC12 cells subjected to OGD. These findings could help enrich our knowledge on the pathogenesis of and provide new therapeutic targets for HIBD.

## Materials and Methods

**Figure 6:**
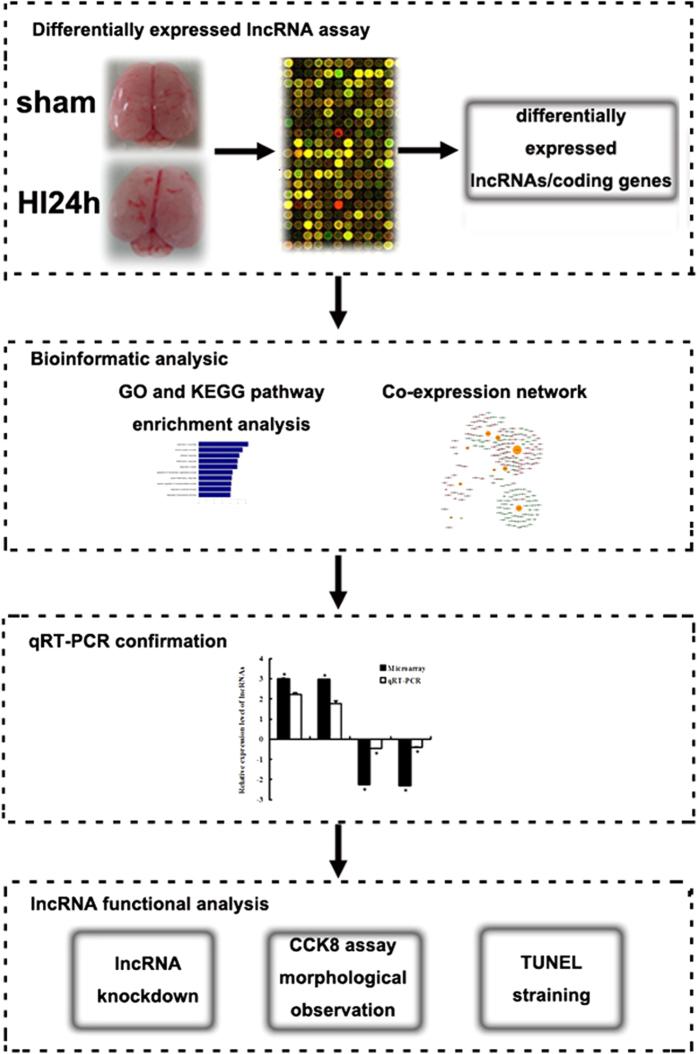
The flowchart of method procedure. The scheme includes four steps. Firstly, differentially expressed lncRNAs and coding genes in neonatal brains following hypoxic-ischemic brain injury compared with sham control brains were identified via microarray analysis. Then, bioinformatics analysis, including GO and KEGG pathway enrichment and CNC network, were performed for speculating the correlation between lncRNAs and coding genes. Subsequently, we utilized qRT-PCR to validate the microarray results. Finally, lncRNA function was analyzed by lncRNA knockdown and subsequent CCK8 and TUNEL detection.

The study contained four procedural modules, including differentially expressed lncRNA assay, bioinformatics analysis and functional analysis (Fig. 6).

### Animal protocols

Postnatal day 10 Sprague-Dawley rats (18–20 g, without gender selection) were obtained from Medical Animal Center of Sichuan Province (Chengdu, Sichuan, CN). All animal procedures were approved by Sichuan University Committee on Animal Research, and the methods were carried out in accordance with the approved guidelines. HIBD was induced according to previously described methods^36^. Briefly, after anesthetization with ether, rats were subjected to ischemia, with the right common carotid artery permanently double ligated and sliced from the middle. After recovery for 1 h, the animals were exposed to hypoxia (8% O_2_, 92% N_2_) for 2.5 h and then returned to their cage. Sham control rats were subjected to isolation and stringing of vessels without occlusion and subsequent ischemia. At 24 h after the HI insult, the rats were sacrificed, and their ipsilateral hemispheres—consisting of both the cortex and hippocampus—were collected for follow-up experiments.

### RNA isolation and labeling

Total RNA (plus lncRNA) was extracted from brain samples (containing both cortex and hippocampus) using TRIzol Reagent (Invitrogen) according to the manufacturer’s protocol. The concentration and quality of RNA was assessed via NanoDrop ND-1000 spectrophotometry (Thermo Scientific) and denatured for agarose gel electrophoresis. After removal of rRNA, purified RNA was amplified and transcribed into fluorescent cRNA using Agilent’s Quick Amp Labeling protocol according to the manufacturer’s protocol.

### Microarray analysis

Microarray analysis was performed by KangChen Bio-tech, Shanghai, PR China. Briefly, the labeled cRNAs were hybridized onto the 4 × 44 K Rat LncRNA Array (Arraystar, Rockville, MD) at 65 °C for 17 h. Hybridization images were collected using an Agilent Microarray Scanner G2565BA. Data were analyzed using Agilent Feature Extraction software. Further analysis was performed using the GeneSpring GX v11.5.1 software package (Agilent Technologies).

### Gene ontology analysis

Gene ontology (GO) analysis provides a controlled vocabulary to describe gene and gene product attributes in any organism ( http://www.geneontology.org). This ontology covers three domains: biological processes, cellular components and molecular functions. Fisher’s exact test was used to detect overlap between the differentially expressed list and the GO annotation list beyond that which would be expected by chance. The *P* value denotes the significance of GO term enrichment among differentially expressed genes (*P* value ≤ 0.05 is recommended).

Pathway analysis is used to map genes to KEGG pathways. The *P* value (EASE-score, Fisher *P* value or hypergeometric *P* value) denotes the significance of the pathway correlations (*P* value ≤ 0.05 is recommended).

### Coding noncoding gene co-expression network

To explore the relationship between lncRNAs and mRNAs, we constructed a coding/non-coding gene co-expression network using 6 mRNAs and the differentially expressed lncRNAs. We calculated the Pearson correlation coefficient (PCC) and used the *R*-value to calculate the correlation coefficient of the PCC between lncRNAs and coding genes (lncRNA-coding PCC, not including lncRNA-lncRNA or coding-coding PCC). We subsequently screened based on the Pearson correlation coefficient using the selection parameter PCC ≥ 0.90 as meaningful. We illustrated the co-expression network using Cytoscape (v2.8.1). Analyses were performed by Kangchen Bio-tech (Shanghai, P.R. China).

### Quantitative real-time PCR (qRT-PCR)

Total RNA was extracted from brain samples (including both cortex and hippocampus) using TRIzol reagent (Invitrogen) and converted into cDNA using the Fermentas RT kit according to the manufacturer’s instructions. PCR was performed in a total reaction volume of 25 μL, containing 12.5 μL SYBR Premix Ex Taq (2×), 2 μL cDNA, 1 μL forward primer (10 μM), 1 μL reverse primer (10 μM), 0.5 μL ROX Reference Dye II (50×), and 8 μL double-distilled water. The amplification conditions were as follows: 10 min at 95 °C to initiate denaturation; 40 cycles of 5 s at 95 °C, 30 s at 63 °C, and 30 s at 72 °C; and a final extension for 5 min at 72 °C. Amplification efficiency was evaluated via standard curve analysis. All samples were normalized to GAPDH, and the experiment was repeated three times.

### Cell culture

Rat phenochromocytoma PC 12 cells were cultured in Dulbecco’s modified Eagle’s medium (DMEM, Gibco) supplemented with 10% fetal bovine serum (FBS, Gibco) on collagen I-coated dishes at 37 °C in 5% CO_2_/95% air.

### Oxygen glucose deprivation (OGD) models

After washed twice with PBS, PC12 cells were incubated in glucose-free DMEM and subsequently exposed to hypoxia (95% N_2_/5%CO_2_). After 6 h, cells were returned to the normoxic incubator with FBS and glucose-supplemented DMEM and incubated fro another 24 h.

### Small interfering RNA (siRNA) transfection

Three different siRNAs (siRNA-1606, siRNA-1447, siRNA-3047; Gene Pharma, Shanghai, China) targeted BC088414 were transfected into PC12 cells by X-tremeGENE siRNA transfectcion reagent (Roche, Mannheim, Germany) individually or in combination (named as siRNA-2662). Nonsense siRNA were transfected as negative control (NC), and non-transfected cells as BLANK control. The sequences of the 3 siRNAs were as follows:

siRNA-1606: 5′-GGAAAUCCCUCUAUAAGAATT-3′;

siRNA-1447: 5′-GCUUCUGCUUCCUCACCUUTT-3′;

siRNA-3047: 5′-CCAAUCCACUGAACGCCAATT-3′.

The inhibition efficiency of siRNA was tested by qRT-PCR.

### Cell viability assays

Cell viability was evaluated by Cell counting Kit (CCK8, Dojindo, Kumamoto, Japan) according to the manufacturer’s introduction. Briefly, cells were plated on 12-well plates. 24 h after OGD, CCK8 solution were added to each cell and then incubated at 37 °C for 3 h. Optical densities were measured at 450 nm by a microplate reader (Biotec, USA).

### TUNEL staining

Cell apoptosis were detected by TUNEL assay with the *in situ* cell death detection kit (Roche, Germany) following the manufacturer’s instruction. Briefly, sections were undertaken hydrogen peroxide treatment and permeailization, then treated with TUNEL reaction mixture at 37 °C for 1 h, followed by mounted with DAPI for 5 min. images were visualized using a fluorescence microscope (Leica DM IRB Olympus BX61). Ten fields were chosen randomly to count apoptotic and total cells at 400× magnification. The apoptotic index (AI) was calculated as follows: AI = (number of apoptotic cells/ total number counted) ×100%.

## Additional Information

**How to cite this article**: Zhao, F. *et al.* Microarray Profiling and Co-Expression Network Analysis of LncRNAs and mRNAs in Neonatal Rats Following Hypoxic-ischemic Brain Damage. *Sci. Rep.* **5**, 13850; doi: 10.1038/srep13850 (2015).

## Figures and Tables

**Table 1:** Numbers of lncRNAs and mRNAs differentially expressed between HI 24 h and sham animals.

	**Upregulated**	**Downregulated**	**Total**
LncRNA			
Fold change > 1.5	157 (48.8%)	165 (51.2%)	322
Fold change > 2.0	54 (65.9%)	28 (34.1%)	82
mRNA			
Fold change > 1.5	215 (57.3%)	160 (42.7%)	375
Fold change > 2.0	44 (54.3%)	37 (45.7%)	81

**Table 2:** Validation of microarray results by qRT-PCR.

**LncRNA ID**	**Microarray**		**qRT-PCR**	
	**Fold change**^a^	***P*** **value**^b^	**Fold change**^a^	***P*** **value**^b^
BC088414	1.675	0.005	2.522	0.083
XR005906	2.976	0.005	1.779	0.115
BC085355	−2.253	0.004	−0.457	0.006
XR006102	−2.304	0.005	−0.402	0.005

^1^Values indicate the absolute fold-change between the groups (HI 24 h to sham ratio) detected by microarray or qRT-PCR; positive values refer to upregulation, and negative values refer to downregulation.

^2^P value was calculated by the student’s T-test (paired).
